# High Expansion Auxetic Skin Graft Simulants for Severe Burn Injury Mitigation

**DOI:** 10.3390/ebj4010011

**Published:** 2023-03-11

**Authors:** Vivek Gupta, Gurpreet Singh, Arnab Chanda

**Affiliations:** 1 Centre for Biomedical Engineering, Indian Institute of Technology (IIT), Delhi 110016, India; 2 Department of Biomedical Engineering, All India Institute of Medical Sciences (AIIMS), Delhi 110029, India

**Keywords:** biaxial testing, tissue simulants, stress analysis, auxetics, skin grafts

## Abstract

Burn injuries are commonly treated with split-thickness skin grafting. However, low expansions offered by spilt-thickness skin grafting inhibit the treatment of large and severe burn injuries when limited donor skin is available. To overcome this gap, in this work, it was attempted to study the expansion potential of skin grafts with novel auxetic incisions with rotating rectangle (RR), honeycomb (HC), alternating slit (AS), H-shaped (HS), Y-shaped (YS), and I-shaped (IS) unit cells, through development of skin graft simulants. Clinically relevant biaxial load testing was conducted to estimate the stress–strain response, void area, and meshing ratio. Moreover, hyperelastic constitutive models were employed to characterize the non-linear biomechanical behavior of the skin graft simulants. The maximum void area increase was observed in the HS skin graft simulant, indicating low skin cover. Overall, the IS auxetic skin graft design exhibited meshing ratio higher than traditional grafts (>3:1), low void area and stresses, which can be beneficial for large skin cover and burn wound healing. With further optimization and clinical tests, the auxetic skin graft designs may find a place with the graft manufacturers for fabrication of grafts with better surgical outcomes for severe burn injuries.

## 1. Introduction

Every year, over 7,400,000 people suffer from burns all over the world [1,2,3]. While nearly two-thirds of burns are moderate and easily recoverable, severe and large burns are difficult to treat and need the support of skin grafts to heal timely [2,3]. The donor area is limited and insufficient to cover a large burn area through traditional skin grafting techniques [4,5]. Skin grafting is a surgical operation in which a small piece of healthy skin is grafted onto damaged skin [4,6]. The limited amount of donor skin availability is one of the most significant challenges associated with skin grafting. Burn wounds have the potential to induce severe bleeding, dehydration, and infection if a transplant is not performed [7,8]. With less donor skin requirement, split thickness skin grafting (STSG) is the most commonly used procedure for covering burn areas [7]. The partial dermis and the entire epidermis layer of skin are harvested for use in a split-thickness skin transplant procedure. While STSG is successful in most burn procedures, its application in severe burns is limited due to low graft expansions [9,10].

It is essential to perform mechanical testing on soft tissues (such as skin, muscles, brain, kidney, and heart) both prior to and after the diagnosis of injuries or disorders [11,12]. Characterizing the mechanical properties of soft tissues would be advantageous in a variety of contexts, including, but not limited to, research on trauma, ballistic testing, surgical training, medical research, finite element modelling, progressive disease modelling, and the simulation of biomechanical behavior in tissue models [12]. Mechanical properties of soft tissues (e.g., stress, deformation, fracture) illustrate the tissue life, behavior, strength, and fracture limits [13,14]. Soft tissues exhibit non-homogeneous, nonlinear, viscoelastic, anisotropic behavior due to oriented collagen fibers [13,15,16]. Biaxial testing machines have been developed and utilized by several researchers in order to describe the mechanical characteristics that soft tissue retains [7,8,9,10,11]. Using an elastomeric material, Xue et al. [17] created a tunable valve and investigated its mechanical properties. They made anisotropic scaffolds and then computed the biaxial stress using different cross-fiber orientations. Androulidakis et al. [18] designed a device for conducting experiments in which two-dimensional materials are put through a controlled equibiaxial strain on supported beams. These beams can be bent either upwards or downwards to put the material under compressive or tensile loadings, respectively. The biaxial mechanical properties of an untreated and glutaraldehyde-treated aortic valve cusp were investigated by Billiar et al. [19]. They noticed that when exposed to equibiaxial strain, a strong mechani-cal link between the biaxial stretch axis caused the creation of negative circumferential stresses. The anisotropic fiber-reinforced glycosaminoglycan hydrogels were created by Ravishankar et al. [20] for the development of the heart valve. In their research, the combination of hydrogels and fibres produced robust, reinforced composites that imitate the mechanical performance of heart valves while retaining high cell survival and favorable activation and proliferation indicators. In traditional skin grafting method, topology optimization can increase the meshing ratio. Sutula et al. [21] included phasefields into topology optimization for maximizing compliance. Phasefields were regarded as an efficient model for estimating the onset of fractures or damage, and optimal designs were proposed for skin graft patterns, which may be utilized to determine the optimal parameters for higher expansion in a certain design. Due to very high expansions produced by auxetic polymers, it was hypothesized that these patterns applied to skin grafting would be able to generate expansions significantly greater than those produced by conventional split-thickness skin grafts, without inducing stresses greater than the ultimate tensile stress of skin. Sacks et al. [22] studied the mechanical properties of several biological materials through development of a biaxial testing device and mechanical testing.

In the literature, several research works on the mechanical properties of skin are reported [12,23]. Chanda et al. [15] studied the mechanical properties of human skin and compared them with skin simulants. In their study, they developed tissue simulants that exhibited properties similar to the skin, which can be used for surgical training. Gupta et al. [9] designed and developed oval shape skin graft simulants using the additive manufacturing (AM) method. They studied the mechanical properties of these skin graft simulants with a varying spacing and size. To date, while a lot of research has been conducted in characterization of skin mechanics and development of skin simulants, limited works have focused on characterization of skin grafts and their expansions. Recent experimental evaluation of SGSTs have indicated significant differences between the expansion or meshing ratio (ratio of the area covered and initial area of the graft), claimed by the skin graft mesher manufacturers, and the actual meshing ratios [24,25]. Gupta et al. [9] also observed a similar trend through computational and experimental analysis of traditional skin graft simulants. Recently, incisions with innovative auxetic structures have been tested computationally on skin grafts, revealing good expansion potentials [3,26]. Based on these extensive works, the hypothesis of our study is that experimental expansion potential of skin grafts with auxetic incisions would be much higher than that of traditional skin grafts.

## 2. Materials and Methods

The current work aims to develop high-fidelity skin graft simulants with auxetic incisions, characterize their expansions, and compare them with that of traditional skin grafts. In this study, a custom made planar biaxial tester was employed to test the auxetic skin graft simulants biaxially with clinically relevant loads. Six novel graft simulant designs with different auxetic structures were fabricated using a set of innovative steps, including additive manufacturing. A patented skin simulant material [15] was used to develop the non-linear skin graft simulants, and a range of hyperelastic curve fit models were employed to characterize their biomechanical responses. Expansion was measured in terms of meshing ratios and void areas and were comparably with that of traditional skin grafts.

### 2.1. Design and Fabrication of Auxetic Skin Graft Simulants

**Figure 1:**
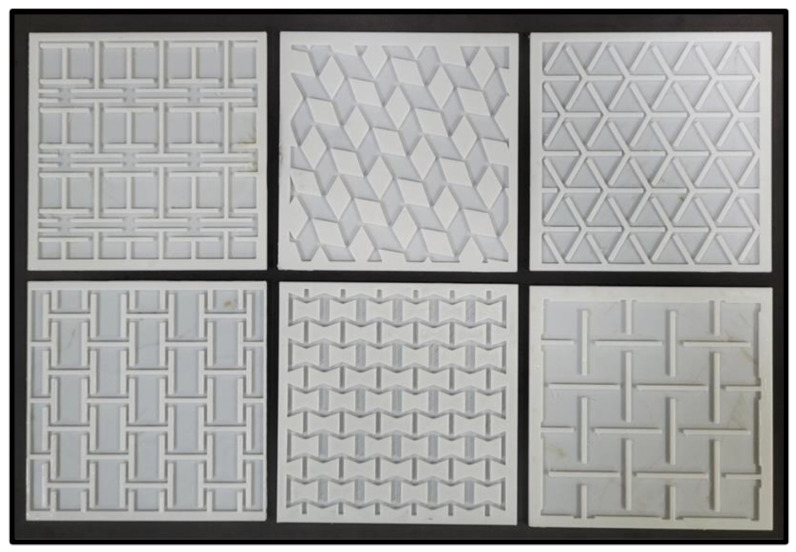
Three-dimensionally printed casting molds of skin graft models.

The designs of these auxetic structures were selected based on the negative Poisson’s ratio and the high expansion potential of auxetic structures reported in previous studies [27]. SolidWorks was used to design the molds of auxetic skin grafts and exported in the .stl format. The outer dimension of each mold was 100 mm × 100 mm × 2 mm and 5 mm offset in each direction with different auxetic structures. Figure 1 shows the molds of different auxetic patterns: I-shaped, RR-shaped, Y-shaped, H-shaped, honeycomb-shaped, and AS-shaped. Similar to the previous studies [9], these mold designs were used to fabricate the auxetic skin graft molds using an additive manufacturing technique.

**Figure 2:**
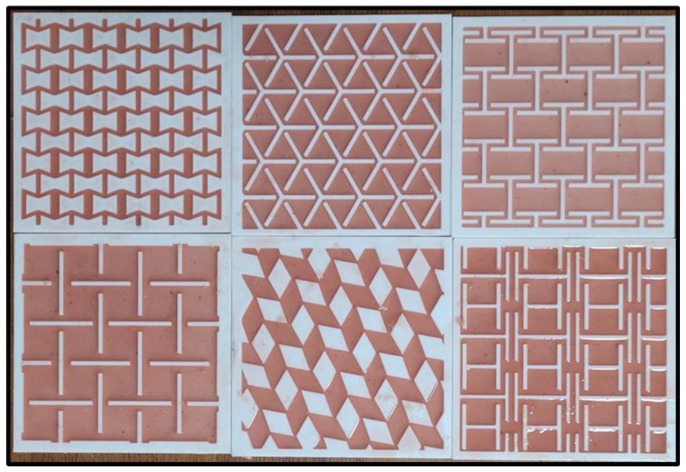
Three-dimensionally printed molds with silicone.

The two-part polymeric material was used to mimic the mechanical properties of skin. Based upon the previous studies, two-part polymeric material was mixed properly with equal proportion (1:1 by weight) [26]. It was poured and left to cure for 6–7 h, and after completing the curing time, skin graft simulants were removed from the molds and used for the testing. Figure 2 shows the skin graft simulants with a maximum size of 100 mm × 100 mm × 2 mm. The developed skin graft simulants mimic the shore hardness of skin which was 15 A ± 1 A. The fabricated auxetic skin graft simulants were tested on a planer biaxial testing machine.

### 2.2. Design and Development of Planar Biaxial Tester

**Figure 3:**
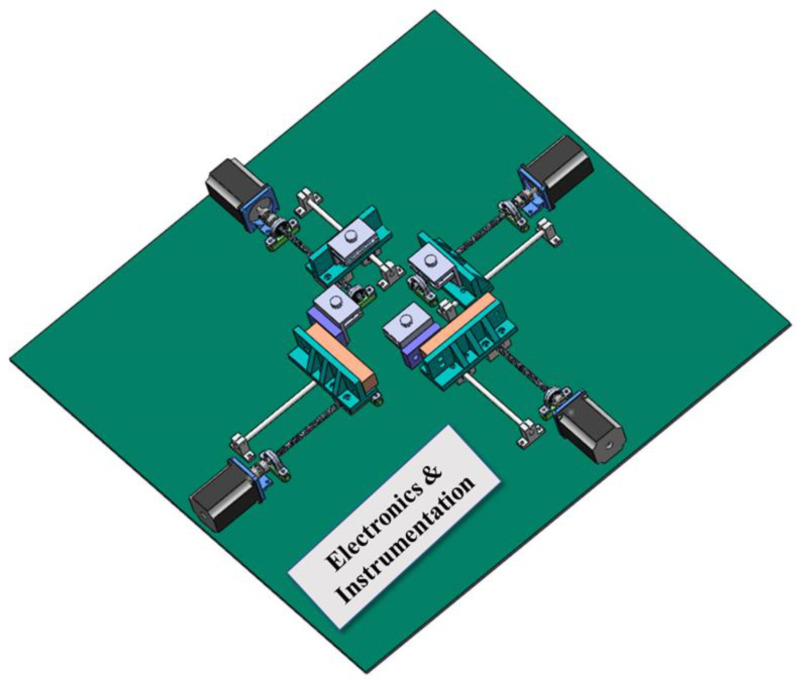
Three-dimensional schematic diagram of planer biaxial tester.

An innovative and low-cost planer biaxial device is designed and developed to test auxetic skin graft patterns. An 8-bit microcontroller was used to provide axial motion and force calculation. This device has the capacity to test samples up to 0.4 kN load with different speeds. Moreover, the applied displacement and forces are displayed on LCD, and stored the force-displacement data in the system (i.e., Microsoft Excel). Figure 1 shows the schematic representation of the planer biaxial tester. It has mainly three sections: gripping, axial movement, electronics, and instrumentation (E&I). A gripper is used to hold the sample tightly without any damage. For axial movement, motors were connected with loadcell and leadscrew. In the electronics section, Arduino was connected with loadcell and motors. These are the three main building blocks of the planer biaxial tester device (Figure 3). 

The system was developed to optimize the size and dynamically control speed. A steeper motor, coupler, lead screw, lead screw nut, 3D printed parts, aluminum rod, and linear bearing make up the motion mechanism. Three-dimensionally-printed item attached to clamps and lead screw nut. The 3D-printed assembly had a load cell. Motor speed was changed to see how it affected results. The stepper motor calculates up to 16th parts every rotation; hence, the minimum step distance is 0.5 mm, and the speed is 30 mm/min. The minimum and maximum loads that can tested on the implemented load cells were 10 gm (i.e., approximately 0.1 N) and 32 kg (i.e., approximately 320 N), respectively. In addition to this, load cell had a rated output of 1.0 ± 0.15 mV/V and a zero output of 0 ± 0.1 mV/V. The overall input impedance of the loadcell was 1115 ± 10% Ω.

**Figure 4:**
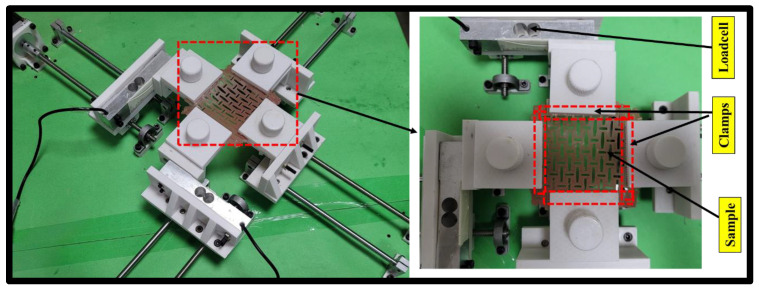
Sample attachment with 3D-printed clamps.

Figure 4 illustrates the gripper (clamp) connected to the 3D-printed component and the load cell schematically. This gripper was designed and made using 3D printing to hold the sample tightly with friction to avoid slippage during the testing. 3D printed clamps with flat pyramids on their surface were utilised in a prior work by Scholze et al. [28] for the stretching of tissues like skin, ligaments, and tendons. It was discovered that this design efficiently holds and stretch the soft tissue samples at high fatigue cycles. A clamp with lateral blocks and asymmetrical tooth jaws was used to test the soft issues in another study by Shi et al. [29]. It was discovered that the suggested design could test the samples up to 6.87 kN without slipping. Moreover, it was observed that the clamps may stretch the soft tissue samples to the point of rupture. Another 3D printing-based clamp was reported by Wood et al. [30] to investigate the mechanical characteristics of soft tissues at low stresses. The tissue samples were held using clamps with multiple sleeves for proper gripping. To hold the sample firmly and damage-free, 3D-printed nuts and bolts were fastened to the cantilever portion of the gripper.

In the cantilever section of the gripper, 3D-printed nuts and bolts were attached to hold the sample tightly without any damage. The required gripping force to attach the sample tightly was minimal and depended on the soft tissue, the sample tested, and the test condition. For testing the skin graft simulants, a biaxial tester was used. The sample was attached with all four clamps tightly. Two loadcells were used to calculate the force data, which were attached to the simulant samples. The stress (σ) and strain (dε) values were calculated using Equations (1) and (2), respectively.

(1) $$\mathrm{Stress}\;(\sigma )=\frac{Equivalent\;Force\;(F)}{Cross\;-\;section\;area\;(A)}$$

(2) $$\mathrm{Strain}\;(d\varepsilon )=\frac{Change\;in\;length\;(dL)}{Initial\;length\;(L)}$$

The E&I measures force and displacement. Arduino, load cell, LCD, HX711, and A4988 IC were E&I system components. The load cell was immediately attached to 3D-printed clamps for measuring force in X and Y directions with a maximum capacity of 40 kg, sufficient for soft tissue characterization. The load cell works on the basis of the wheat stone bridge. Calibrated with known weights and set to zero, a minor variation in voltage was reflected in force. A common HX711 24-bit amplifier was used to visualize the slight voltage shift. One end of the load cell was attached to the 3D-printed item, and the other to the clamps. The sample stretches as the motor moves, increasing force. LCD displays X and Y force values. A programmable microcontroller can save load cell force values as.csv files. Arduino connects hardware and software. It helps manage lead screw motion and measure sample force at a specified strain rate. The results can be viewed on a computer or smartphone using a controller and LCD display. MS Excel or Matlab (MathWorks, Carlsbad, CA, USA) can plot the findings.

### 2.3. Material Modelling 

Soft tissue and polymer-based materials have a non-linear stress–strain profile. Skin grafts have been observed to have less stress compared to the skin at high strain [31,32]. Hyperelastic constitutive material models (e.g., Fung, Yeoh, Humphrey) can be used to characterize the non-linear mechanical behavior of skin graft simulants. In this study, Yeoh, Mooney–Rivlin, and Neo-Hookean hyperelastic curve fit models are used to calculate the constant coefficients ($c_{1}$, $c_{2}$, and $c_{3}$). Non-linear hyperelastic curves work on the strain energy density function (Ѱ), which is based on the material type. In an isotropic hyperelastic model, the strain-energy function is dependent on the principal stretches (*λ_1_, λ_2_,* and *λ_3_*) or the Cauchy-green tensor invariants (*I*_1_, *I*_2_, and *I*_3_, which are also functions of the principal stretches) as shown in Equation (3)–(6).

(3) $${Ѱ}_{isotropic}=Ѱ(I_{1},I_{2},I_{3})$$

(4) $$I_{1}=\sum_{i=1}^{3}\lambda_{i}^{2}$$

(5) $$I_{2}=\sum_{i,j=1}^{3}\lambda_{i}^{2}\lambda_{j}^{2}\;\;i\neq j$$

(6) $$I_{3}=\prod_{i=1}^{3}\lambda_{i}^{2}$$

The strain energy and stress function of hyperelastic models were shown in Equations (7)–(13)

(7) $${Ѱ}_{Yeoh}=c_{1}{\left(I_{1}-3\right)}^{1}+c_{2}{\left(I_{1}-3\right)}^{2}+c_{3}{\left(I_{1}-3\right)}^{3}$$

(8) $${Ѱ}_{Mooney-Rivlin}=c_{1}(I_{1}-3)+c_{2}(I_{2}-3)$$

(9) $${Ѱ}_{Neo-Hookean}=c_{1}(I_{1}-3)$$

(10) $$\sigma_{1}=\lambda_{1}\frac{\partial \Psi }{\partial \lambda_{1}}-\lambda_{3}\frac{\partial \Psi }{\partial \lambda_{3}}$$

(11) $$\sigma_{Yeoh}=2(\lambda^{2}-\frac{1}{\lambda })(c_{1}+{2c}_{2}(I_{1}-3)+{3c}_{3}{\left(I_{1}-3\right)}^{2})$$

(12) $$\sigma_{Mooney-Rivlin}=2(\lambda^{2}-\frac{1}{\lambda })(c_{1}+c_{2}\frac{1}{\lambda })$$

(13) $$\sigma_{Neo-Hookean}=2(\lambda^{2}-\frac{1}{\lambda })(c_{1})$$

The meshing ratio was calculated at 100% strain using Equation (14). The maximum void area in the unit call was calculated computationally using SolidWorks. At 100% strain, the maximum void area was calculated using an image and this exercise was repeated three times. These results will help and guide the surgeon to choose the skin grafts based on the burn region.

(14) $$\mathrm{MR}=\frac{Expanded\;Area}{Unexpanded\;Area}$$

## 3. Results and Discussions 

### 3.1. Deformation Analysis of Auxetic Skin Graft Simulants 

**Figure 5:**
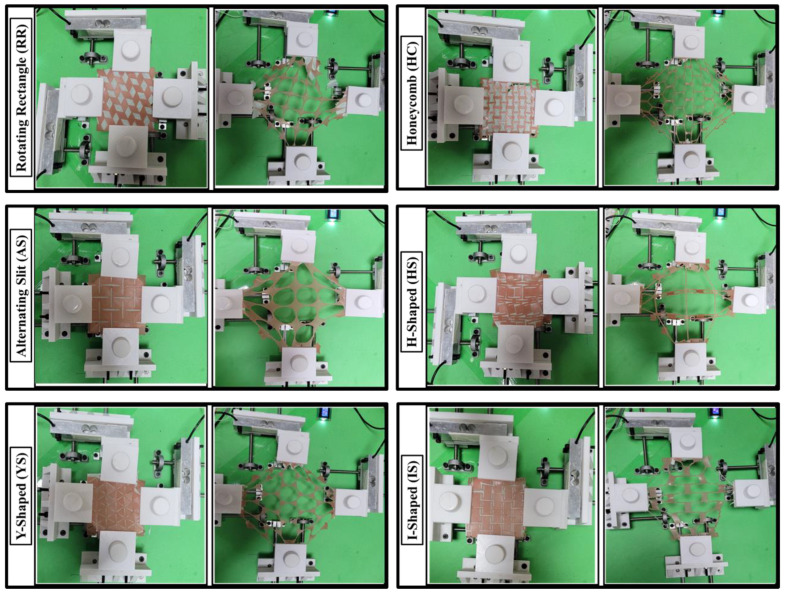
Initial and final deformation of the auxetic skin graft simulants using biaxial testing.

Figure 5 illustrates the biaxial expansion of different auxetic skin graft simulants at 0 and 100% strain. It was observed across all the figures that the maximum void area of unit cells was increased with strain (Table 1). In RR skin graft simulants, the structure was changed into the oval voids with less amount of linkage (14 linkages) between unit cells. The final void area was 4.3 times higher than the initial design at 100% strain. In order to simulate HC-shaped skin grafts, the structure was changed to include hexagonal gaps connected by links (26 linkages) between the unit cells. At 100% strain, the resulting void area was 3.6 times larger than that calculated at the start. For AS-shaped skin graft simulants, the structure was transformed into circular voids (16 voids) with a substantial amount of intercellular connection. At 100% strain, the final void area was 9.6 times larger than the initial design. In HS skin graft simulants, the structure was modified into four large hollow square voids. The final void area at 100% strain was 9.3 times higher than the design. The structure of the YS-shaped skin graft simulants was modified to include significant circular gaps and star-shaped linkages between the unit cells. At 100% strain, the finished void area was 6.4 times larger than the one designed at the beginning. For I-shaped skin graft simulants, the structure was changed into the rectangle shape voids with a high amount of linkage between unit cells. The final void area was 5.5 times higher than the initial design at 100% strain.

### 3.2. Stress Analysis of Auxetic Skin Graft Simulants 

**Figure 6:**
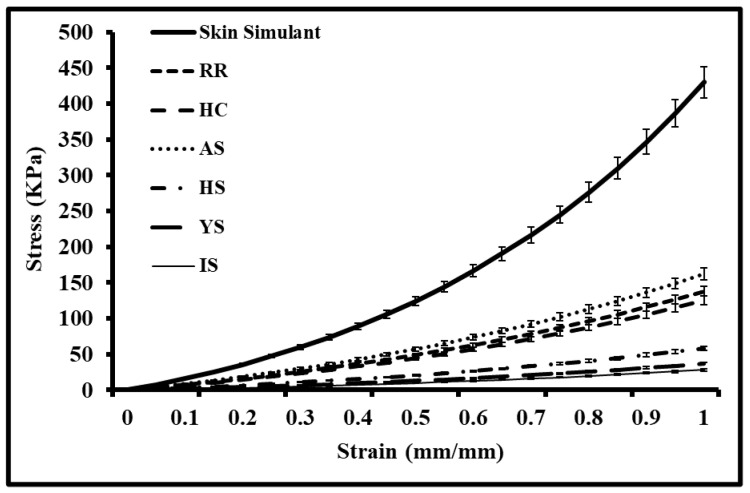
Stress–strain analysis of different auxetic skin graft simulants up to 100% stretch.

Figure 6 illustrates the equivalent stress–strain analysis of different auxetic skin graft simulants using a planer biaxial testing machine. The force-displacement data were converted into the stress–strain using Equations (1) and (2). All the skin graft simulants were tested up to 100% strain or the stretch of 2. The minimum and maximum stress induced was found in IS and AS-shaped skin graft simulants. The maximum induced stress shows a high chance of graft rupture. The stress–strain graph was helpful for understanding the strength and failure of the skin graft simulants. From all designed skin graft simulants, minimum induced stress (28 KPa) was chosen to be the non-breakable grafts (ultimate tensile strength) for this study.

### 3.3. Meshing Ratio Analysis of Auxetic Skin Graft Simulants

**Figure 7:**
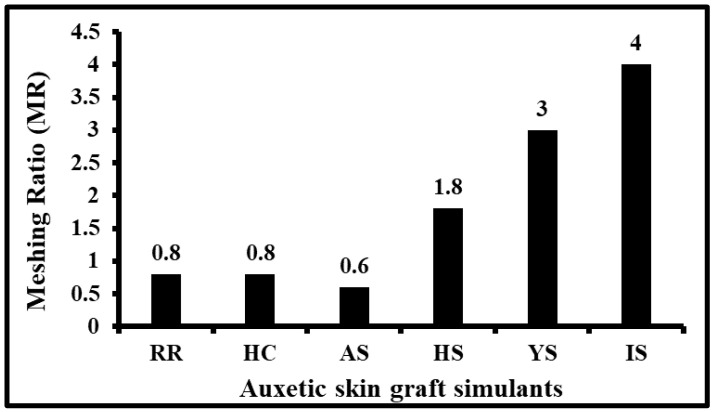
Maximum meshing ratio of different auxetic skin graft simulants at control value.

The maximum possible expansion without possible AS-shaped skin graft rupture was estimated for all dimensional parameters under biaxial tests. Figure 7 illustrates the possible meshing ratio (MR) at control values. All the MR values were calculated at maximum induced stress (i.e., at 100% of IS). In IS skin graft model, the maximum MR at 100% strain was 4. YS skin graft simulants show the maximum MR value of 3 at 80% strain, which is 25% less than the maximum MR value at the same stress level. For HS skin graft simulants, the maximum estimated MR value was 1.8 at the maximum induced stress level. These MR values were 55% and 40% lesser than IS and YS skin graft simulants, respectively. Similarly, for other skin graft models. The minimum MR values were observed in AS-shaped skin graft simulants at the control value. 

### 3.4. Hyperelastic Non-linear Curve Fits of Auxetic Skin Graft Simulants

**Figure 8:**
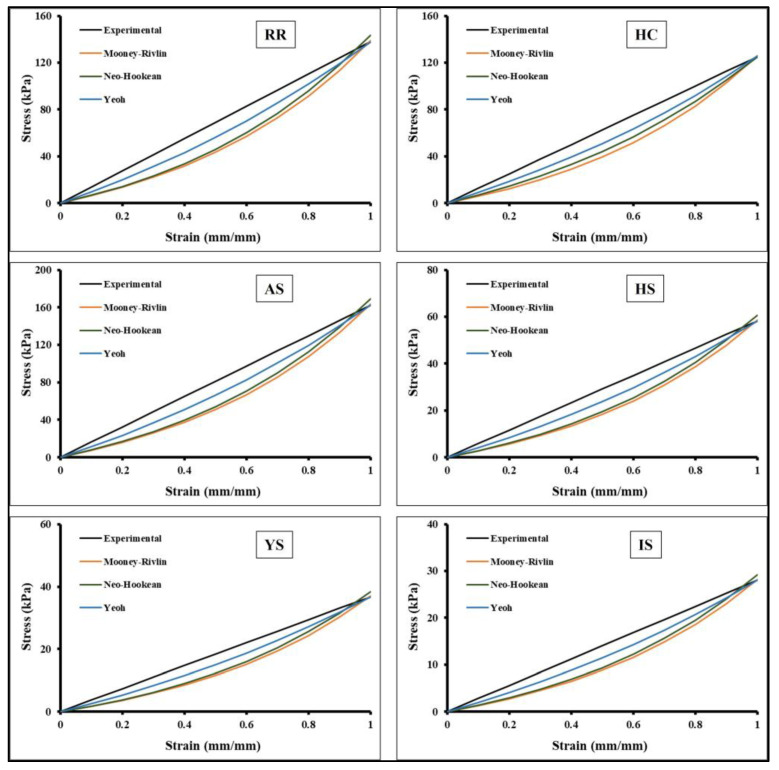
Different hyperelastic curve fit models with experimental results of auxetic structures.

Hyperelastic curve fit standards were used to characterize the stress and stretch response of the skin graft simulants, as discussed in the materials and methods section. The average of five stress versus strain results of skin graft simulant tested at 24 mm/min strain rate are shown in Figure 8. These hyperelastic curve fit models were used to fit the constant coefficients similar to previous studies [9]. Table 2 shows the constant coefficients of Mooney–Rivlin, Neo-Hookean, and Yeoh non-linear hyperelastic models. The accuracy of prediction of the non-linear behavior of auxetic skin graft simulants was estimated for each hyperelastic model (Table 2). Table 2 summarizes the coefficients and R^2^ values, which fall within 0.90 < R^2^ < 1. For the Mooney–Rivlin model, the value of constant coefficients varied from 2.23 to 16.87. The maximum and minimum value was found in the AS and I-shaped skin graft simulants, respectively. The value of the constant coefficients in the Neo-Hookean model was found to range from around 4.17 to 24.15. The AS and I-shaped skin graft simulants were found to have the highest and lowest values, respectively, when compared to one another skin graft designs. The range of possible values for the constant coefficients in the Yeoh model was from 4.75 to 27.50. Both the highest and lowest values were discovered in the skin graft simulants that were shaped like AS and IS, respectively. Overall, the hyperelastic curve fit coefficients were used to study the computational models. 

## 4. Discussion

This work aimed to design and develop different auxetic skin grafts simulants. A 3D modelling software was used to design the auxetic patterns and additive manufacturing was employed to fabricate molds of these auxetic skin graft simulants. A mixture of two-part polymeric material was poured into these molds to develop the skin graft simulants. These skin graft simulants were tested on a custom-built biaxial testing device to study the biomechanical properties and relative expansion of the novel graft models.

Under biaxial displacements, the unit cell deformation of each structure was different. In the literature, similar structural change results were observed computationally in alternating slit, I-shaped, and Y-shaped patterns [33,34,35]. Recently, in 2022, Singh et al. [26] developed and studied rotating triangle shaped skin graft simulants under uniaxial loads, and characterized the structural changes, expansions, and mechanical properties. The study concluded that the skin graft simulants show expansion potential higher than traditional skin grafts. However, no study to date has conducted biaxial testing of skin grafts computationally or experimentally with clinically relevant loads. To overcome these challenges, the current study focused on conducting biaxial mechanical experiments on novel auxetic skin graft simulants [26,33,34,35].

The maximum biaxial stress induced in all auxetic skin graft simulants was below that of the skin simulant. The I-shaped skin graft simulant showed the minimum stress across all the auxetic skin graft simulants. A similar computational study was performed by Gupta et al. [3] with different I-shaped auxetic skin graft designs. In their study, the maximum and minimum von Mises stresses were observed in I-shaped skin graft models, consistent with our findings.

These results show the relative meshing ratios of different auxetic structures when stretched up to 100% strain, which is anticipated to provide the insights into expansions that these structures can exhibit when stretched up to the ultimate tensile stress of skin. Annaidh et al. [23] studied the mechanical properties of human skin at different body locations. They observed that ultimate tensile strength was different for skin at each body location and reported a mean value of 21.6 ± 8.4 MPa.

While calculating the meshing ratio, 100% strain was chosen to effectively compare the auxetic skin graft simulants. These relative results are anticipated to provide the insights into expansions which these structures can exhibit when stretched up to the ultimate tensile stress of skin. It was observed that I-shaped auxetic skin graft simulant shows the maximum meshing ratio. Across all the auxetic skin graft simulants, the observed meshing ratio was higher than that of traditional skin grafts.

In past studies, Capek et al. [4] uniaxially studied the influence of Langer lines and orientation on skin grafting. They observed that when the Langer lines changed from perpendicular to parallel, maximum meshing ratio decreased by up to 20%. Pripotnev et al. [7] conducted an experimental study on 210 burn patients. In all the patients, the burn area of more than 20% of the total body surface was considered. Kan et al. [36] conducted a similar study with skin grafting experiments on 51 burn patients with different meshing ratios of 1.5:1, 3:1, and 6:1. They observed that none of the experiment studies reached the claimed meshing ratio and maximum meshing ratio value was 3.38 in 6:1 skin grafts. Recently, studies reported that auxetic skin graft structures could be used for the large wound to cover a maximum area with minimal donor skin [37]. Additionally, not only is meshing utilized for treating burned skin, but it also has other applications. It can be utilized for the replacement of large amounts of wounded skin as well as any other function. Overall, from this study, the I-shaped design shows relatively higher expansion than other models, and much higher than traditional skin grafts.

## 5. Conclusions

In this study, skin graft simulants were developed with novel auxetic incisions to study their expansion potential. A biofidelic skin simulant was developed and casted using 3D printing techniques to generate the graft simulants. A planer biaxial tester was used to test the auxetic skin graft simulants with clinically relevant loads. The stress–strain responses, void areas, and meshing ratios were estimated for up to 100% stretch, for all graft simulants, and compared. Moreover, hyperelastic material modeling was conducted to characterize the biomechanical properties of the skin graft simulants. From the results, the I-shaped auxetic skin graft design exhibited meshing ratio higher than traditional grafts (>3:1), low void area and stresses, and was concluded to be a good candidate for further clinical studies, and for generating large skin cover and expedited wound healing.

There are some limitations of this study that should be acknowledged. Firstly, isotropic skin graft simulants were employed for the study of skin grafts expansion. Secondly, the thickness of all the skin graft simulants was considered to be constant to maintain consistency of the results. Thirdly, only one strain rate, based on literature, was used to test the skin graft simulants. Fourthly, the human cadaveric skin or animal skin was not implemented, and wound healing was not considered in this study. Additionally, it is important to mention that biological investigations with animal skin need to be conducted in vivo, to estimate cell proliferation and wound healing with the novel auxetic skin graft designs. Furthermore, biological investigations were not conducted to characterize the effect of voids on cells proliferation and wound healing. In future studies, anisotropic cadaveric human skin of varied shapes and thicknesses will be adopted, and a range of strain rates will be tested on the auxetic skin graft simulants to conduct more precise analysis.

In conclusion, the different auxetic skin graft simulants show potential for enhanced biaxial skin graft expansions. A few novel graft designs were identified, which, with further parametric optimization and clinical testing, are anticipated to generate large skin graft cover with limited donor skin, which can lead to ground-breaking improvements in burn surgery outcomes.

## Figures and Tables

**Table 1:** Void area of different skin graft simulants.

Design	Initial Void Area (mm^2^)	Final Void Area (mm^2^)	% Void Area Increase
AS	90	867	863.0
HC	111.4	409.63	267.7
HS	142.49	1613.18	1032.1
IS	135.29	750.27	454.6
RR	116.36	500.62	330.2
YS	122.15	785.86	543.3

**Table 2:** Hyperelastic curve fit coefficients.

Hyperelastic Model	Curve Fit Coefficient	RR	HC	AS	HS	YS	IS
Mooney–Rivlin	*c*_1_	14.34949	13.01418	16.87916	6.065835	3.825152	2.917043
	*c*_2_	10.98502	09.96286	12.92231	4.644426	2.928595	2.233293
Neo-Hookean	*c*_1_	20.53203	18.62142	24.15204	8.679786	5.473409	4.173975
Yeoh	*c*_1_	23.38589	21.22468	27.50868	9.885044	6.233806	4.753983
	*c*_2_	−0.22559	−0.20745	−0.26527	−0.09521	−0.06008	−0.04584
	*c*_3_	0.001536	0.001551	0.001802	0.000641	0.000407	0.000311

## Data Availability

The datasets generated during and/or analyzed during the current study are not publicly available due to large dataset, but are available from the corresponding author on reasonable request.
